# The clinicopathological and prognostic significance of PD-L1 expression in gastric cancer: a meta-analysis of 10 studies with 1,901 patients

**DOI:** 10.1038/srep37933

**Published:** 2016-11-28

**Authors:** Minghui Zhang, Yuandi Dong, Haitao Liu, Yan Wang, Shu Zhao, Qijia Xuan, Yan Wang, Qingyuan Zhang

**Affiliations:** 1 Department of Medical Oncology, Harbin Medical University Cancer Hospital, Harbin 150081, China; 2 Department of Surgical Oncology, Harbin Medical University Cancer Hospital, Harbin 150081, China; 3 Department of Intensive Care Unit, Harbin Medical University Cancer Hospital, Harbin 150081, China; 4 Department of Medical Oncology, Heilongjiang Provincial Hospital, Harbin 150000, China

## Abstract

The prognostic value of programmed death-ligand 1 (PD-L1) in gastric cancer (GC) remains controversial. To clarify this problem, we performed a meta-analysis of research studies identified in the PubMed, EMBASE and the Cochrane Library databases. A total of 1,901 patients in 10 studies were enrolled in this meta-analysis, and the pooled hazard ratio (HR) of 1.64 (95% CI 1.11 to 2.43; P = 0.01) indicated that PD-L1 expression is associated with a shorter overall survival (OS). The pooled odds ratios (ORs) indicated that PD-L1 expression was associated with tumour size (OR = 1.87, 95% CI 1.25 to 2.78; P = 0.002) and lymph node status (OR = 2.17, 95% CI 1.04 to 4.52; P = 0.04). However, PD-L1 had no correlation with gender, age, cancer location, differentiation, depth of invasion, and tumour stage. This meta-analysis indicates that PD-L1 expression is a valuable predictor of the prognosis of patients with GC. PD-L1 expression could be used for identifying a subgroup of patients, who would potentially benefit from targeted therapy against PD-1 or PD-L1. Well-designed large-cohort studies are needed to confirm these findings.

Gastric cancer is the one of the most common cancers around the world^1^. The majority of patients are diagnosed at an advanced stage, with 50%–75% presenting with regional lymph node metastasis, and the overall five-year survival rate of advanced stage GC patients is approximately 15%^2^. Although multimodality therapy has advanced, the prognosis and overall survival rate of patients with gastric cancer are still low^3^. Thus, to improve the prognosis of such patients, novel strategies need to be developed and established.

Programmed death 1 (PD-1), which belongs to the CD28 family, is a receptor expressed on the surface of activated T cells that regulates their proliferation and activation^4^. PD-L1 (also known as B7-H1) is the major ligand for PD-1 and is expressed in some tumour cells as well as by activated B cells and T cells, dendritic cells, myeloid cells, and endothelial cells^5^. The binding of PD-L1 to PD-1 leads to apoptosis or exhaustion in activated T cells^6^. PD-L1 expression is induced by tumour cells via variable mechanisms, thereby helping tumour cells escape from immune surveillance^7^. In addition, PD-L1 has a pivotal role in the conversion of naive T cells to regulatory T cells (Tregs) by inhibiting the Akt-mammalian target of rapamycin (mTOR) signalling^8^. The expression of PD-L1 has been correlated with poor clinical outcomes in several human cancers^9^,^10^,^11^,^12^. Given this background, a PD-1/PD-L1 pathway blockade by targeted-antibodies (against PD-1 or PD-L1) is a highly promising therapy and has elicited durable antitumor responses and long-term remission in recent clinical trials^13^.

Data on the prevalence and prognostic significance of PD-L1 expression in GC is very limited and remains controversial. We performed a meta-analysis to assess whether PD-L1 expression was correlated with the clinicopathological features and prognosis of GC patients.

## Results

### Study selection and characteristics

**Figure 1:**
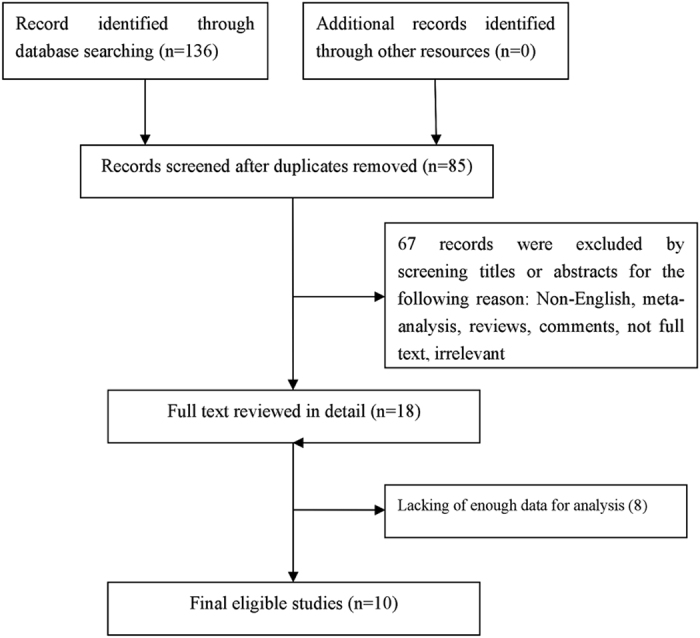
Flow Chart of Study Selection.

In the present study, 136 articles were identified by the initial search strategy. Through reading the study titles and abstracts, 118 records were removed because of duplicate studies and non-relevance with the theme. After we reviewed the full texts of the 18 potentially eligible articles in detail, 10 trials meeting the inclusion criteria were included in the final analysis. A flowchart depicting the study selection is shown in Fig. 1.

The characteristics of the included studies are shown in Table 1 and Supplementary Table S1. In total, 10 studies including 1,901 patients were included in the pooled analysis. OS was recorded in all studies. Study quality, as assessed by the Newcastle-Ottawa quality assessment scale, ranged from six to eight. Hence, the studies were of a relatively high quality.

### PD-L1 as a prognostic factor for GC

**Figure 2:**
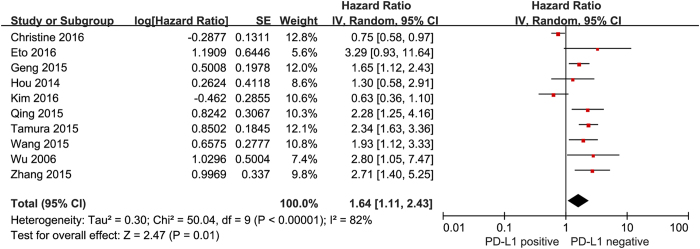
Forest plot describing the association between PD-L1 expression and OS of patients with gastric cancer.

**Figure 3:**
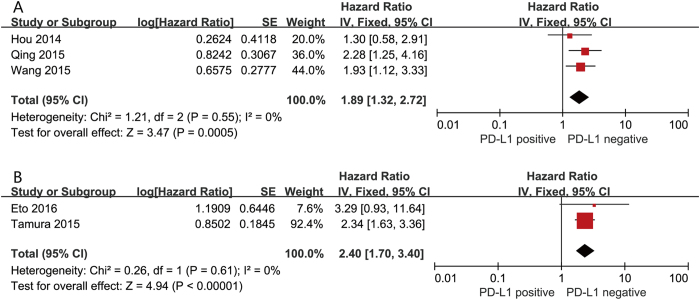
Forest plot describing subgroup analysis of the association between PD-L1 expression and OS according to immunohistochemistry cutoff value. (**A**) Percentage (cutoff value 10%), (**B**) percentage (cutoff value 51%).

We evaluated the correlation between PD-L1 expression and OS among the 1,901 patients from all ten included studies. The pooled HR for OS showed that PD-L1 expression was associated with poor OS (HR 1.64, 95% CI 1.11 to 2.43, P = 0.01) in GC with a 64% increase in the risk for mortality (Fig. 2). In addition, when using the percentage evaluation method, we found numerically inferior survival in the PD-L1 positive group if we took 51% (HR 2.40, 95% CI 1.70–3.40, P < 0.001) as well as 10% (HR 1.89, 95% CI 1.32–2.72, P < 0.001) as the cut-off value (Fig. 3).

### The correlation between PD-L1 expression and clinicopathological features

#### Gender

**Figure 4:**
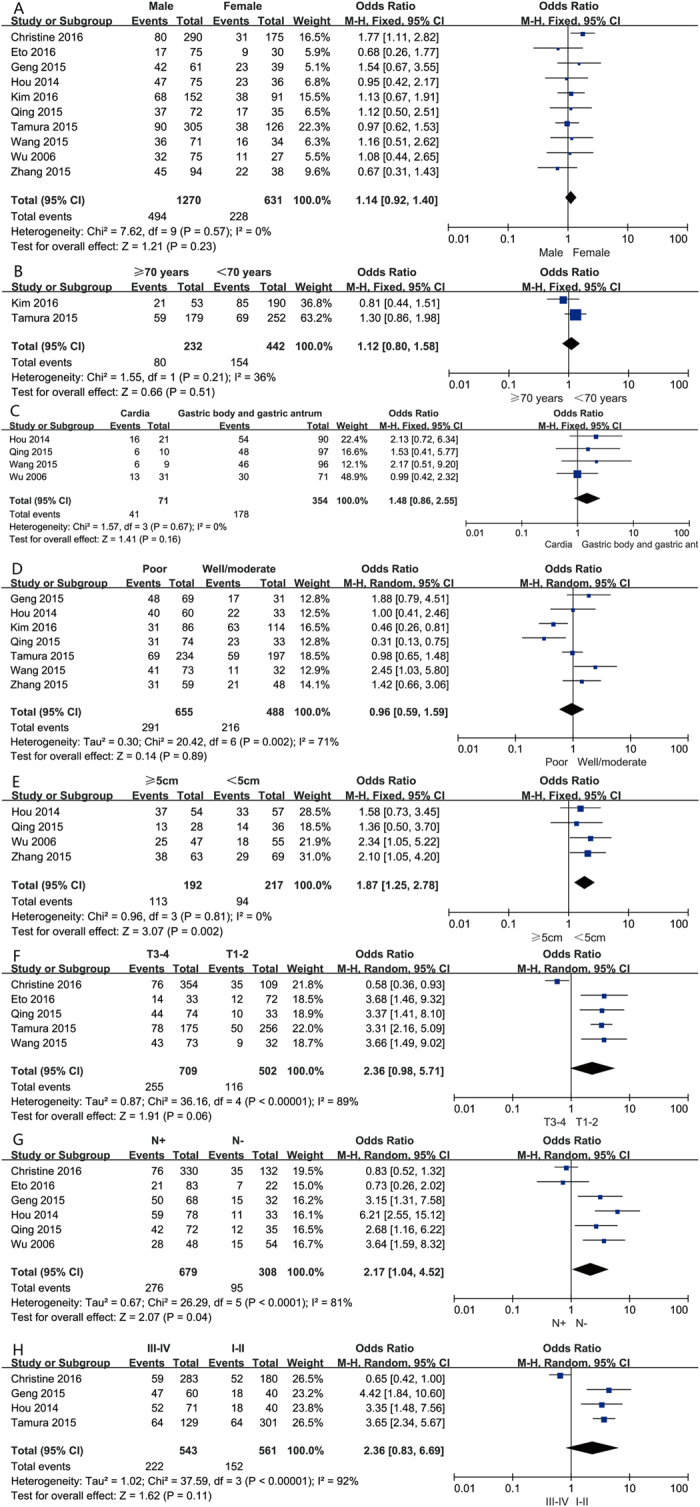
Forest plots for the association between PD-L1 expression and clinicopathological features (**A**) gender, (**B**) age, (**C**) cancer location, (**D**) differentiation, (**E**) tumor size, (**F**) dept of invasion, (**G**) lymph node metastasis, H. stage.

We evaluated the correlation between PD-L1 expression and gender in a panel of 1,901 patients from all ten studies. Of 1,270 male patients, 494 (38.9%) were PD-L1 expression positive, and 228 (36.1%) of 631 female patients were PD-L1 expression positive. The pooled OR indicated no significant correlation between PD-L1 expression and gender (OR = 1.14; 95% CI = 0.92–1.40, *P* = 0.23) (Fig. 4A).

#### Age

The correlation between PD-L1 expression and age was analysed in two studies, including a total of 674 patients. Eighty patients (34.5%) were PD-L1 expression positive, among a total of 232 older patients (≥70 years), and 154 (34.8%) of 442 younger patients (<70 years) were PD-L1 expression positive. No significant association was found between PD-L1 expression and gender (OR = 1.12; 95% CI = 0.80–1.58, P = 0.51) (Fig. 4B).

#### Cancer location

Four out of the ten studies, including 425 patients, examined the relationship between PD-L1 expression and cancer location. Of 71 cardia patients, 41 (57.7%) were PD-L1 expression positive, and 178 patients (50.3%) were PD-L1 expression positive among 354 patients with gastric body and antrum cancers. The pooled OR indicated that PD-L1 expression had no clear correlation with cancer location (Fig. 4C).

#### Differentiation

Seven studies, including 1143 patients, were analysed for the correlation between PD-L1 expression and tumour differentiation. Of 655 poorly differentiated tumours, 291 (44.4%) were PD-L1 expression positive, and 216 (44.3%) of 488 moderately/well-differentiated cases were PD-L1 expression positive. No significant association was found between PD-L1 expression and tumour differentiation (OR = 0.96, 95% CI 0.59 to 1.59, P = 0.89) (Fig. 4D).

#### Tumour size

The relationship between PD-L1 expression and tumour size was evaluated in four studies including 409 patients. One hundred and thirteen (58.9%) patients were PD-L1 expression positive out of a total of 192 patients with large tumours (≥5 cm), and 94 (43.3%) out of a total of 217 patients with small tumours (<5 cm) were PD-L1 expression positive. Increased PD-L1 expression was found to be significantly associated with large tumour size (OR = 1.87, 95% CI 1.25 to 2.78, P = 0.002) (Fig. 4E).

#### Depth of invasion

Five studies with a total of 1,211 patients were analysed for the relationship between PD-L1 expression and the depth of tumour invasion. Positive PD-L1 expression was found in 255 (36%) out of 709 T3–4 level tumour invasion patients, while 116 (23.1%) out of 502 T1–2 level tumour invasion patients were PD-L1 expression positive. No significant relationship was detected between PD-L1 expression and the depth of tumour invasion (OR = 2.36, 95% CI 0.98 to 5.71, P = 0.06) (Fig. 4F).

#### Lymph node metastasis

Six studies comprising 987 patients were evaluated for the correlation between PD-L1 expression and lymph node metastasis. Of 679 patients with positive lymph node metastasis, 276 (40.6%) were PD L1 expression positive, and 95 (30.8%) of 308 patients with negative lymph node metastasis were PD-L1 expression positive. The combined OR for the positive lymph node metastasis group versus the negative lymph node metastasis group was 2.17 (CI 1.04 to 4.52, P = 0.04) (Fig. 4G).

#### Stage

Four studies, including 1104 patients, were analysed for the association between PD-L1 expression and TNM stage. Two hundred and twenty-two (40.9%) of 543 stage III–IV patients were PD-L1 expression positive and 152 (27.1%) out of 561 stage I-II patients were PD-L1 expression positive. No significant association was found between PD-L1 expression and stage (OR 2.36, 95% CI 0.83 to 6.69; P = 0.11) (Fig. 4H).

Heterogeneity was observed in the analysis of PD-L1 expression with histological differentiation (P = 0.002; I^2^ = 71%), the depth of tumour invasion (P < 0.001; I^2^ = 89%), lymph node status (P < 0.001; I^2^ = 81%), and tumour stage (P < 0.001; I^2^ = 92%). Thus, a random-effects model was employed for the analysis. The other analyses above were carried out using a fixed effects model.

### Publication bias and sensitivity analysis

**Figure 5:**
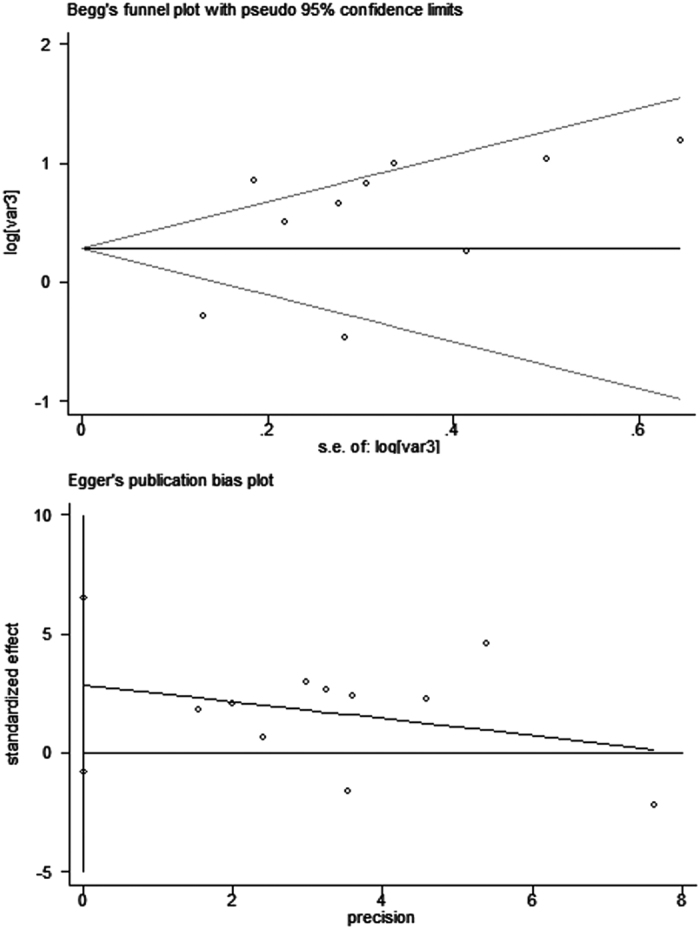
Egger’s and Begg’s funnel plot with 95% confidence intervals for OS publication bias testing.

**Figure 6:**
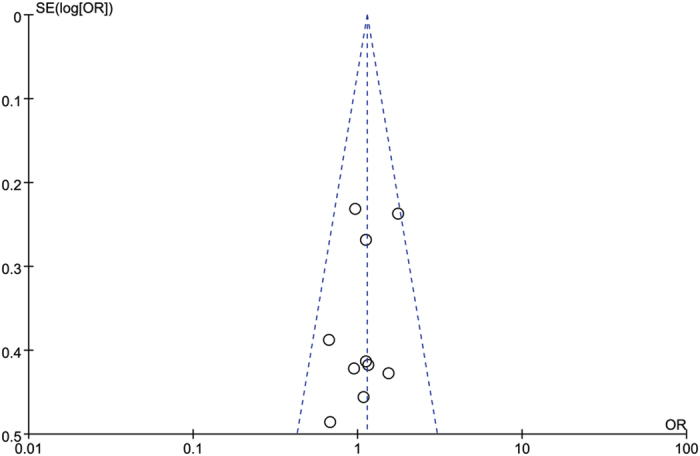
Funnel blot was designed to visualize a potential publication bias for PD-L1 expression and gender.

Egger’s and Begg’s test indicated no publication bias among these studies regarding the hazard ratio and overall survival, with P values of 0.109 and 0.592, respectively (Fig. 5). Visual inspection of the funnel plots revealed no publication bias for gender (Fig. 6). Publication bias was not investigated when the number of studies was less than 10 because of the low sensitivity of the qualitative and quantitative tests^14^.

A sensitivity analysis, in which one study was removed at a time, was performed to evaluate the stability of our results. The results demonstrated that no individual study significantly influenced the overall HRs. This suggested that the results of the present meta-analysis are credible.

## Discussion

PD-L1 overexpression has been observed in various solid tumours, and several studies have demonstrated that the expression of PD-L1 plays a key role in cancer immune escape and the associated tumour progression and poor prognosis^15^. These highlighted studies demonstrated that PD-L1 may serve as a potential prognostic and predictive biomarker. However, for patients with GC, the association between the expression of PD-L1 and their prognosis remains controversial. Multiple studies have indicated that positive PD-L1 expression is associated with a significantly poor OS^16^,^17^,^18^,^19^,^20^,^21^,^22^,^23^, but other studies could not confirm this finding^24^,^25^.

In the present meta-analysis, we pooled all available data from published studies to evaluate the correlation between PD-L1 expression and GC prognosis. Our results suggest that the up-regulation of the expression of the PD-L1 protein contributes to the poor prognosis of GC. In 2015, Huang *et al*.^26^ also analysed the prognostic value of PD-L1 in gastrointestinal tract cancer. This study demonstrated that positive PD-L1 expression was a negative predictor for OS. Another recent meta-analysis also found that patients with PD-L1-positive expression had significantly shorter survival time compared with the PD-L1-negative group in East Asia. These results are consistent with our study. However, this meta-analysis did not include non-Asian populations, and did not present the correlation between PD-L1 expression and tumour size, lymph node status, gender, age, cancer location, differentiation, depth of invasion, and tumour stage^27^. In addition, our study strictly screened the literature according to the inclusion criteria. To better analyze the correlation between PD-L1 expression and the clinical parameters, the inclusion criteria clearly require that the original article must provide two or more clinical parameters. Thus, Sun *et al*.^28^ did not meet the inclusion criteria and was excluded.

Based on our results, we consider PD-L1 overexpression to be a risk factor and a new biomarker for the prediction of GC prognosis. There are some possible explanations for the correlation between PD-L1 expression and a poor prognosis. First, the engagement of PD-L1 and PD-1 may induce activated T cell apoptosis, exhaustion, and interleukin-10 (IL-10) expression^4^. Second, PD-L1 may function as a molecular shield to protect PD-L1 positive tumour cells from CD8+ T cell–mediated lysis^29^. Third, PD-L1 can promote the generation of induced Tregs by down-regulating the mTOR, AKT, S6 and the phosphorylation of ERK2 and increases PTEN, thus restraining the activity of effecter T-cells^8^. These actions of PD-L1 expression on the tumour can promote T-cell tolerance and escape host immunity. In addition, due to the lack of uniform cut-off values, we conducted relative subgroup analyses. The pooled subgroup results showed that studies using 51% as the cut-off value had a greater difference in OS betweenPD-L1 positive and negative groups than those using 10%, and the HRs were 2.40 and 1.89, respectively.

Biomarker-driven selection of immunotherapy responders improves therapeutic efficacy, minimizes unnecessary exposure and reduces the financial burden on health systems. Recent studies show that high levels of PD-L1 expression are associated with higher clinical activity in patients with various cancer types treated with PD-1/PD-L1-targeted therapy^30^. In our study, we investigated the relationship between the expression of PD-L1 and clinicopathological factors. According to our pooled analysis, patients with larger tumours and positive lymph node metastasis tend to have higher levels of PD-L1 expression. These patients may benefit more from treatment targeting the PD-1/PD-L1 pathway. Additionally, larger tumours and positive lymph node metastasis are also associated with an advanced stage and a poor prognosis, and our results provide a scientific rationale and direct support for the current clinical application of anti-PD-1/PD-L1 immunotherapy in patients with advanced GC that otherwise lack effective treatment options. Our study also indirectly proves that high levels of PD-L1 protein expression are associated with poor GS prognosis. However, another recent meta-analysis showed that increased PD-L1 expression was significantly associated with a poorer tumour stage but not with a larger tumour size^31^, which is inconsistent with our study. This discrepancy may be explained by the number of articles included in the mate-analysis: we included 10 articles while the previous meta-analysis included^5^.

Efforts were made to conduct a comprehensive analysis, but there are limitations to our study. First, the cut-off values distinguishing positive and negative PD-L1 expression determined by IHC varied in different studies, which might cause heterogeneity among the overall results. The subgroup results should have addressed some of these concerns. Second, the distinct antibodies of PD-L1 expression among diverse studies might also impact the accurate estimation of the prognosis for gastric cancer. Standardized methods and definitions of PD-L1 positivity are clearly needed to facilitate studies of PD-L1 as a prognosis biomarker. Hence, a large multicenter study using the same antibody and cutoff for PD-L1 expression may be helpful to obtain more accurate results. Third, not all of the HRs with 95% CI was directly extracted from the studies, so we based our study on the data extracted from Kaplan-Meier curves, which compromises the precision of the data. Fourth, most of the eligible studies failed to provide data regarding progression free survival, so we only extracted OS data in our meta-analysis. Fifth, this meta-analysis was limited to articles published in English, possibly resulting in a publication bias. Despite the above limitations, this meta-analysis demonstrates the correlation between PD-L1 expression and the clinicopathological factors of GC. The results may lead to improvements in the outcomes of anti-PD-1/PD-L1 therapy due to this convenient stratification method.

In conclusion, our result indicated that PD-L1 overexpression is related to a poor prognosis, large tumours and the presence of lymph node metastasis. This information might prove to be helpful in screening candidates for anti-PD-1/PD-L1 therapy. Well-designed large cohort studies are needed to confirm these findings.

## Methods

### Literature search

Two authors (Zhang MH and Liu HT) independently carried out a comprehensive systematic search of published articles using the PubMed, EMBASE, and Cochrane databases, and any discrepancy was resolved by mutual discussion. The deadline for included articles was April 2016. The following keywords were used: (“PD-L1” or “B7-H1” or “CD274” or “Programmed Cell Death 1 Ligand 1 Protein) and (“gastric cancer” or “gastric neoplasms” or “stomach neoplasms”) with the limit “human”. Additional searches through Google Scholar and a manual search through the reference lists of relevant reviews were also performed.

### Eligibility criteria

The criteria for inclusion were set out as the following: (1) All patients were histologically confirmed as GC; (2) PD-L1 expression was measured in GC tissue using immunohistochemistry; (3) Studies provided the correlation between PD-L1 and overall survival; (4) These studies provided a correlation between PD-L1 and clinicopathological features, including at least two parameters; (5) Studies provided sufficient information to estimate HR about OS; (6) Articles published as a full paper in English. Studies that failed to meet the inclusion criteria were excluded. When duplicate publications were identified, only the newest or most informative single article was selected.

### Data extraction and quality assessment

The data were extracted independently by two reviewers (Zhang MH and Liu HT), and any disagreements between the two reviewers were resolved by consensus involving a third reviewer (Dong YD). The following information was extracted from each included trial: name of the first author, year of publication, country, number of patients, TNM stage, IHC evaluation method, antibody, cut-off, PD-L1-positive expression, clinicopathological parameters and the hazard ratios (HRs) and 95% confidence intervals (CIs) for OS. If the HRs were not directly reported, we contacted the authors of the primary studies for additional data. If the authors did not respond, we extracted data from survival curves.

A quality assessment was independently conducted for all of the included studies by two investigators (Wang Y and Zhao S) using the Newcastle–Ottawa Quality Assessment Scale (NOS), and any disagreements were resolved by discussion and consensus. The NOS comprises the following three parameters of quality: selection, comparability and outcome assessment. The maximum possible score is nine points, and NOS scores greater than six are considered indicative of high-quality studies^32^.

### Statistical methods

Hazard ratios (HRs) and their 95% CIs were combined to measure the effective value. If HRs and corresponding 95% CIs were not available, we calculated these data points from available numerical data using the methods reported by Parmar *et al*.^33^. Data from the Kaplan-Meier survival curves were read using Engauge Digitizer version 4.1. For the pooled analysis of the correlation between PD-L1 expression and the clinicopathological parameters, ORs and their 95% CIs were combined to obtain the effective value. Statistical heterogeneity was evaluated using the chi-squared test and I^2^. Statistically significant heterogeneity was defined as a chi-squared P value < 0.1 or an I^2^ statistic >50%. If heterogeneity was observed, we used a random-effects model to reduce the impact of heterogeneity on the results. If heterogeneity was not observed, a fixed-effects model was used. The potential publication bias was assessed by Egger’s and Begg’s tests. All of the statistical analyses were performed using Review Manager Version 5.3 (Revman the Cochrane Collaboration; Oxford, England) and STATA version 12.0 (Stata Corporation; College Station, TX, USA). P values < 0.05 were considered to indicate statistical significance. All P values and 95% CIs were two-sided.

## Additional Information

**How to cite this article**: Zhang, M. *et al*. The clinicopathological and prognostic significance of PD-L1 expression in gastric cancer: a meta-analysis of 10 studies with 1,901 patients. *Sci. Rep.* **6**, 37933; doi: 10.1038/srep37933 (2016).

**Publisher's note:** Springer Nature remains neutral with regard to jurisdictional claims in published maps and institutional affiliations.

## Supplementary Material

Supplementary Table S1

## Figures and Tables

**Table 1:** Characteristics of the studies included in the meta-analysis. NO. = number of patients, NA = not available.

Author	Year	Country	No.	Stage	IHC evaluation method	Antibody				Cut-off	PD-L1 positive (%)	Outcome	HR estimation	Quality Assessment (score)
						company	source	type	clone					
Wu	2006	China	102	I-IV	Percentage of positive cells and staining intensity	Shuzhou university, China	NA	MAB	2H11	NA	43/102 (42.2)	OS	HR and 95% CI 2.80 (1.05–7.48)	7
Geng	2015	China	100	I-IV	Percentage of positive cells and staining intensity	Novus, USA	NA	MAB	2H11	H-score≥ 3	65/100 (65)	OS	HR and 95% CI 1.65 (1.16–2.73)	7
Hou	2014	China	111	I-IV	Percentage of positive cells	Abcam, UK	Rabbit	PAB	NA	≥10%	70/11 (63.1)	OS	Survival curves 1.30(0.58–2.94)	7
Kim	2016	Japan	243	I-III	Percentage of positive cells and staining intensity	Abcam, UK	Rabbit	PAB	NA	≥10% amd Moderate or strong staining	106/243 (43.6)	OS	Survival curves 0.63(0.36–1.09)	7
Qing	2015	China	107	I-III	Percentage of positive cells	GeneTex, USA	NA	PAB	NA	≥10%	54/107 (50.5)	OS	Survival curves 2.28(1.25–4.16)	6
Tamura	2015	Japan	431	I-IV	Percentage of positive cells	Abcam, Japan	Rabbit	PAB	NA	≥51%	128/431(29.6)	OS	HR and 95% CI 2.34 (1.63–3.37)	7
Wang	2015	China	105	NA	Percentage of positive cells	Gene Tex, USA	NA	PAB	NA	≥10%	52/105 (49.5)	OS	HR and 95% CI 1.93 (1.12–3.32)	6
Zhang	2015	China	132	II-III	Staining intensity	Abcam, UK	Rabbit	PAB	ab58810	Moderate or intense staining	67/132 (50.8)	OS	Survival curves 2.71(1.40–5.23)	7
Christine	2016	German	465	I-IV	Percentage of positive cells and staining intensity	CellSignaling, USA	Rabbit	MAB	E1L3N	IRS>2	140/465(30.1)	OS	HR and 95% CI 0.75 (0.58–0.97)	8
Eto	2016	Japan	105	II-III	Percentage of positive cells	Abcam, UK	Rabbit	MAB	ab174838	≥51%	26/105 (25)	OS	Survival curves 3.29(0.93–11.63)	7
