# Supplementary material for: The clinicopathological and prognostic significance of PD-L1 expression in gastric cancer: a meta-analysis of 10 studies with 1,901 patients
Source: Sci Rep. 2016 Nov 28;6:37933. doi: 10.1038/srep37933 (PMC5124943; doi:10.1038/srep37933)
Supplement: Supplementary Table S1 [file srep37933-s1.doc]

**Article title**: The clinicopathological and prognostic significance of PD-L1 expression in gastric cancer: a meta-analysis of 10 studies with 1,901 patients

**Author names and affiliation**: Minghui Zhang^1+^, Yuandi Dong^2+^, Haitao Liu^3+^, Yan Wang^4^, Shu Zhao^1^, Qijia Xuan^1^, Yan Wang^1^, Qingyuan Zhang^1*^

1 Department of Medical Oncology, Harbin Medical University Cancer Hospital, Harbin150081, China
**2** Department of Surgical Oncology, Harbin Medical University Cancer Hospital, Harbin150081, China

**3** Department of Intensive Care Unit, Harbin Medical University Cancer Hospital, Harbin150081, China

**4** Department of Medical Oncology, Heilongjiang Provincial Hospital, Harbin150000, China
***Correspondence to:** Qingyuan Zhang, **e-mail:** zhma1965210@163.com

+these authors contributed equally to this work

Supplementary Table S1 **Characteristics of the studies included in the meta-analysis.**

| Author | Year | No. of tumor metastasis | No. of tumor non-metastasis | No. of stage I-II | No. of stage III-IV | No. of lymph node metastasis | No. of lymph node non-metastasis |
| --- | --- | --- | --- | --- | --- | --- | --- |
| Wu | 2006 | NA | NA | NA | NA | 48 | 54 |
| Geng | 2015 | NA | NA | 40 | 60 | 68 | 32 |
| Hou | 2014 | NA | NA | 40 | 71 | 78 | 33 |
| Kim | 2016 | 0 | 243 | NA | NA | 124 | 119 |
| Qing | 2015 | NA | NA | NA | NA | 72 | 35 |
| Tamura | 2015 | 42 | 388 | 301 | 129 | 241 | 190 |
| Wang | 2015 | NA | NA | NA | NA | NA | NA |
| Zhang | 2015 | NA | NA | NA | NA | NA | NA |
| Christine | 2016 | 90 | 373 | 180 | 283 | 330 | 132 |
| Eto | 2016 | 0 | 105 | NA | NA | 83 | 22 |

**NO.** =Number, **NA**= not available.
